# Dinuclear Lanthanide Compound as a Promising Luminescent Probe for Al^3+^ Ions

**DOI:** 10.3390/molecules27248761

**Published:** 2022-12-10

**Authors:** Zhi Chen, Yinghao Xie, Zhanbo Li, Tao Lin

**Affiliations:** 1 College of Chemistry and Environmental Engineering, Shenzhen University, Shenzhen 518071, China; 2 College of New Materials and New Energies, Shenzhen Technology University, Shenzhen 518118, China

**Keywords:** lanthanide dinuclear compound, luminescent probe, aluminum

## Abstract

Luminescent probes have wide applications in biological system analysis and environmental science. Here, one novel luminescent dinuclear europium compound with a crown ether analogous ligand was synthesized through a solvent–thermal reaction. Through transformation, upon the addition of Al^3+^ ions to the *N*,*N*′-dimethyl formamide solution of the europium compound, the luminescent intensity of the characteristic emission of Eu^3+^ decreased, and a new emission peak appeared at 346 nm and increased rapidly. The luminescent investigation indicated that it could act as a highly sensitive and selective luminescent probe for Al^3+^ ions. Moreover, mass spectrometry and single-crystal X-ray diffraction confirmed the formation of a new more stable trinuclear aluminium compound during the sensing process.

## 1. Introduction

In past decades, interest in the photophysical properties of lanthanide-based compounds has been strongly stimulated [1,2,3,4]. Benefiting from the narrow characteristic emissions resulting from *f*–*f* transition of lanthanide ions and few perturbations from environments, lanthanide-based compounds have wide applications in biological system analysis and environmental science [5,6,7]. Therefore, lanthanide luminescent probes have become one of the most important methods for sensing ions due to their excellent monochromaticity and high sensitivity [8,9]. Various lanthanide luminescent probes have been reported, which mainly focus on H^+^ [10], F^−^ [11], K^+^ [12], Ag^+^ [13], Ca^2+^ [14], Zn^2+^ [15], Mg^2+^ [16], O_2_ [17], H_2_O_2_ [18], ATP [19], etc.

Moreover, due to the rapid development of metal–organic frameworks (MOFs) [20], several lanthanide metal–organic frameworks (Ln-MOFs) as important luminescent sensors have been widely investigated due to their advantages such as controllable pore sizes, and the diversity of functional organic ligands for interaction recognition [21,22,23,24,25]. However, due to poor solubility, the applications of MOF-based probes are still limited, especially for biologic imaging. Thus, soluble discrete lanthanide metal–organic assemblies would be quite appropriate for this application. However, due to the unpredictable coordination behavior and the lability of the lanthanide coordination bonds, the controllable synthesis of functional polynuclear discrete lanthanide metal-organic assemblies remain challenging [26]. Thus far, relevant reports on discrete polynuclear lanthanide metal–organic assemblies for luminescent probes are still rare [27,28,29,30].

On the other hand, Al^3+^ ions are harmful to the human brain and nervous system, causing Parkinson’s and Alzheimer’s disease [31,32], and they also adversely affect the growth of plants [33]. Thus, the development of a method for the effective detection of Al^3+^ ions is urgent. To date, several chemical sensors based on organic compounds [34,35,36,37,38] and MOFs [39,40,41] for Al^3+^ ion detection have been reported. However, sensors based on discrete lanthanide metal–organic assemblies are rarely reported. In this study, using crown ethers analogous carboxylic ligand 2,2′-(((ethane-1,2-diylbis(oxy))bis(ethane-2,1-diyl))bis(oxy))dibenzoic acid (H_2_TEBA), a novel dinuclear lanthanide compound (**1**, [Na_4_Eu_2_(TEBA)_4_(H_2_O)_4_]·[CuCl_2_]·Cl·H_2_O) was synthesized. Luminescent investigations revealed that it is a promising luminescent probe for Al^3+^ ions. Moreover, the sensing mechanism was studied using mass spectrometry and single-crystal X-ray diffraction and a transformation process was confirmed.

## 2. Results

### 2.1. Synthesis and Structure of Compound ***1***

**Figure 1:**
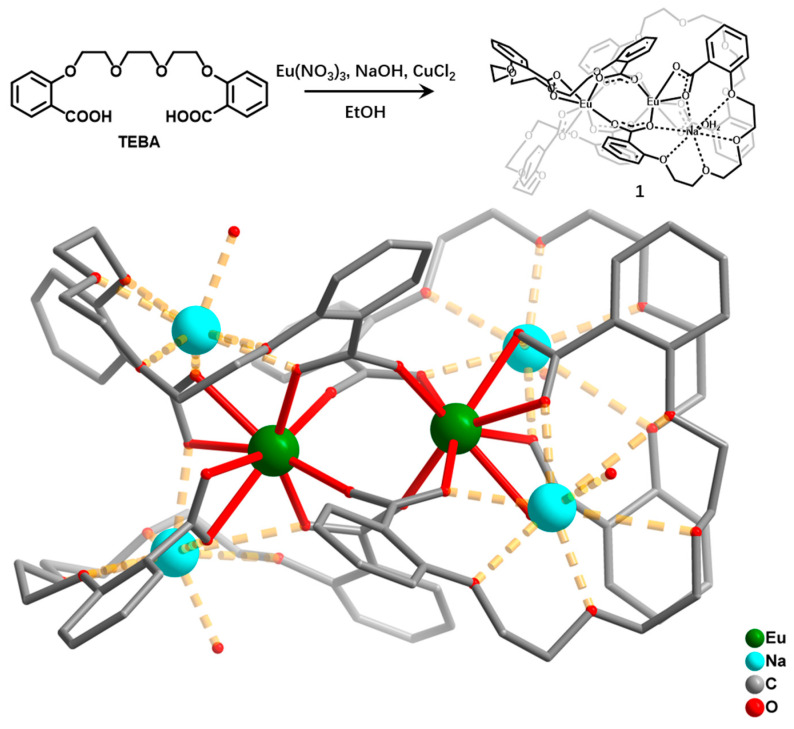
The synthetic route and the molecular structure of compound **1**.

Compound **1** was prepared under solvent-thermal condition. Details of the synthesis are presented in the Materials and Methods. Single-crystal X-ray diffraction analysis revealed that compound **1** belongs to the triclinic *P*$\bar{1}$ space group. The asymmetric unit in compound **1** consists of two dinuclear [Na_4_Eu_2_(TEBA)_4_(H_2_O)_4_]^2+^ units, two [CuCl_2_]^−^ anions, two disorder Cl^−^, and one lattice water (Figure S1). Each Eu^3+^ ion is coordinated by eight O atoms from different TEBA^2−^ ligands. The adjacent two Eu^3+^ ions form a dinuclear unit through the bridges of carboxyl groups from four TEBA^2−^ ligands (Figure 1). The distance between the two Eu^3+^ ions is 4.13 Å. The Na^+^ ion located in the analogous crown ether structure is formed by one TEBA^2−^ ligand, which affords four ether O atoms and two carboxyl O atoms to chelate the Na^+^ ion. Moreover, with one coordinated H_2_O molecule and one carboxyl O atom from another TEBA^2−^ ligand, the Na^+^ ion is eight-coordinated. The Eu–O and Na–O distances are in the range of 2.30–2.58 Å and 2.30–3.01 Å, respectively. The [Cl–Cu–Cl]^−^ anions and the lattice water fill in the space among the [Na_4_Eu_2_(TEBA)_4_(H_2_O)_4_]^2+^ units (Figure S1).

### 2.2. Luminescent and Sensing Properties of Compound ***1***

**Figure 2:**
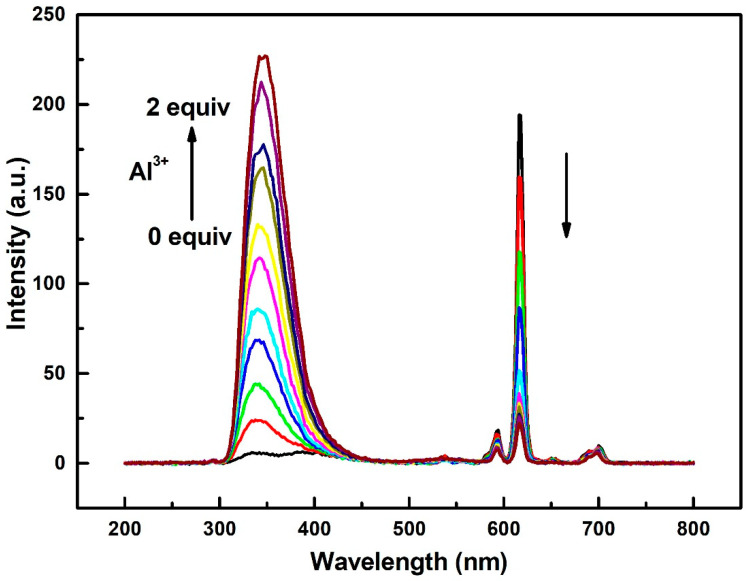
The emission spectra (excited at 292 nm) of compound **1** in DMF at room temperature in the presence of 0 to 2 equiv Al^3+^ ions with respect to compound **1**.

To investigate the luminescent properties of compound **1** in *N*,*N*′-dimethyl formamide (DMF) solution (1 × 10^−4^ mol/L), emission spectrum measurements were performed at room temperature and excited by a UV light with a wavelength of 292 nm. As shown in Figure 2, typical emission peaks of Eu^3+^ ions can be observed, which can be attributed to ^5^D_0_→^7^F_1_ (594 nm), ^5^D_0_→^7^F_2_ (618 nm), and ^5^D_0_→^7^F_4_ (700 nm) transitions (black curve in Figure 2). The luminescent lifetime of the ^5^D_0_→^7^F_2_ transition is 0.33 ms (Figure S3). The intensity of the ^5^D_0_→^7^F_2_ transition (electric dipole) is much stronger than the intensity of the ^5^D_0_→^7^F_1_ transition (magnetic dipole), which indicates that the coordination environment of the Eu^3+^ ion is asymmetric, in agreement with the results from the crystallographic analysis (Figure 1).

**Figure 3:**
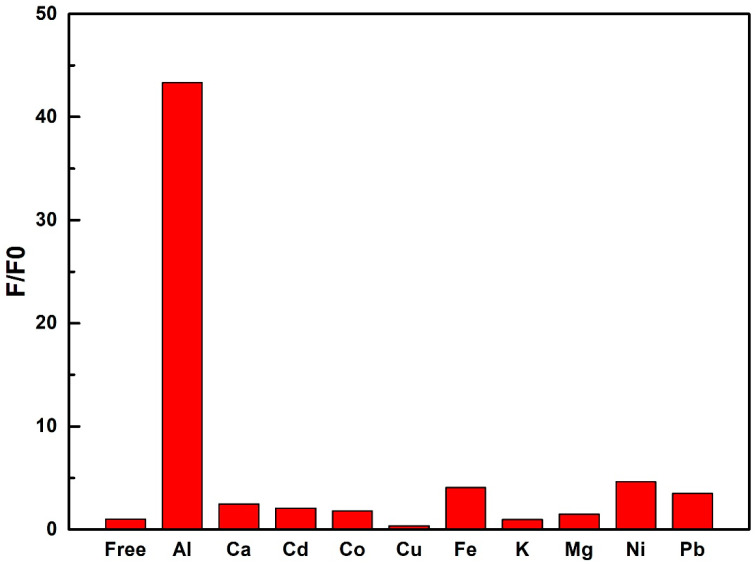
The luminescent intensity of compound **1** in DMF solution at 346 nm upon the addition of 2 equiv of AlCl_3_, CaCl_2_, CdCl_2_, CoCl_2_, CuCl_2_, FeCl_3_, KCl, MgCl_2_, NiCl_2_, and Pb(NO_3_)_2_.

Upon the addition of different cations (Al^3+^, Ca^2+^, Cd^2+^, Co^2+^, Cu^2+^, Fe^3+^, K^+^, Mg^2+^, Ni^2+^, and Pb^2+^) to compound **1** in the DMF solution (1 × 10^−4^ mol/L), the emission intensities of Eu^3+^ ions (such as the peak at 618 nm) become weaker to some extent (Figure 2 and Figure S4). Interestingly, after the addition of Al^3+^ ions to the solution, the emission intensity at 346 nm increases rapidly. Up to 2 equiv Al^3+^ ions with respect to compound **1**, the intensity of the peak at 346 nm becomes around 43 times stronger than that of the original peak. This sensing process containing both a new increasing luminescent peak and a decreasing characteristic emission of Eu^3+^ is a typical OFF-ON and ON-OFF mode. The difference between two peaks is 272 nm, which improves the sensitivity of the sensing process. The emission intensity at 346 nm exhibits a very good linear relationship with the equivalent addition of Al^3+^ ions with a correlation coefficient *r* = 0.999 (Figure S5). When other metal ions were added, there were no significant increase at 346 nm (Figure 3). This implies that compound **1** can determine the concentration of Al^3+^ ions within a certain concentration range. Furthermore, additional sensing characterizations for lower concentrations of Al^3+^ ions were performed to determine the lowest limit of detection (Figure S6). The intensity of emission at 346 nm was almost the same when the concentration of Al ions was below 1 × 10^−6^ M. However, when the concentration of Al^3+^ ions reached 5 × 10^−6^ M, an increase in intensity could clearly be observed. These results show that the detection limit of **1** for sensing Al^3+^ ions was about 5 × 10^−6^ M, exhibiting a good sensitivity for Al^3+^ ions [40].

### 2.3. Sensing Mechanism Studies

The sensing behavior of compound **1** may be attributed to the direct transformation from a compound containing Eu^3+^ to a new compound containing Al^3+^ [42,43,44]. To confirm this suspicion, mass spectrometry and single-crystal X-ray diffraction were performed to verify the new aluminum compound. To investigate the ionic state of compound **1**, electrospray ionization mass spectrometry (ESI-MS) (Figure S7) and matrix assisted laser desorption ionization time-of-flight mass spectrometry (MALDI-TOF MS) (Figure S8) were applied. ESI-MS results for compound **1** in DMF show the main peaks of H_2_TEBA + Na^+^ ([C_20_H_22_O_8_Na]^+^ calcd: 413.12; found: 413.12) rather than the peaks of compound **1** (Figure S7). This can be attributed to the structural destruction of compound **1** due to the high energy of electrospray ionization process. When MALDI-TOF MS was applied, it showed a main fragment [Na_2_Eu(TEBA)_2_]^+^ (C_40_H_40_EuNa_2_O_16_ calcd: 975.13; found: 975.13) from compound **1** (Figure S8). After luminescent intensity of the DMF solution of compound **1** no longer increased upon the addition of Al^3+^ ions, ESI-MS measurements of this solution were performed, and a new *m*/*z* 1261.28 emerged (Figure S9). Compound **1** was destroyed during the ESI-MS measurement and a new peak appeared after the addition of Al^3+^ ions; thus, we speculate that the new peak resulted from a newly formed aluminum compound, which was more stable than compound **1**.

**Figure 4:**
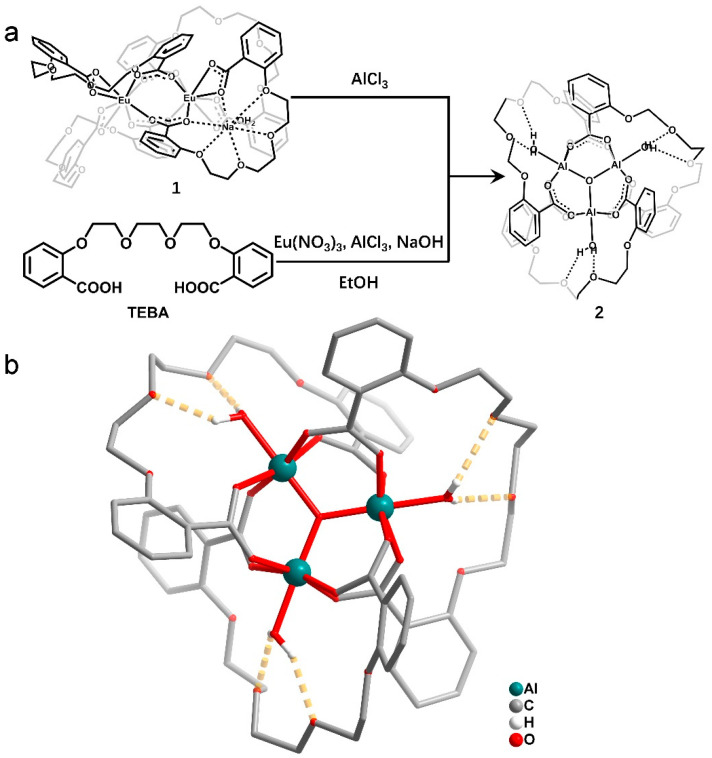
(**a**) The transformation and synthetic paths of compound **2**. (**b**) The molecular structure of compound **2**.

To further determine the origin of the new peak and the structural information of the Al^3+^ compound, we used AlCl_3_·6H_2_O instead of CuCl_2_·2H_2_O under the same synthesis conditions as compound **1**, and colorless long-stripe-like crystals were obtained. Single-crystal X-ray diffraction confirmed that it was a trinuclear aluminum compound with a molecular structure of [Al_3_(*μ*_3_-O)(TEBA)_3_(H_2_O)_3_]_2_·[Eu(NO_3_)_5_]·EtOH·0.5H_2_O (**2**). Compound **2** crystallizes in triclinic *P*$\bar{1}$ space group. Each asymmetric unit consists of two [Al_3_(*μ*_3_-O)(TEBA)_3_(H_2_O)_3_]^+^ units, one [Eu(NO_3_)_5_]^2−^ anion, one lattice disordered ethanol molecule and half a lattice water molecule (Figure S2). The Al^3+^ ion is six-coordinated by four carboxyl O atoms from four different carboxyl groups, one coordinated water molecule and one *μ*_3_-O^2−^ atom, forming an octahedral geometry (Figure 4). Three Al^3+^ ions form a stable trinuclear cluster via the bridge of the *μ*_3_-O^2−^ atom and three TEBA^2−^ ligands. One water molecule coordinates to each Al^3+^ ion, and is located in the center cave of the TEBA^2−^ ligand. Each coordinated water molecule forms two hydrogen bonds with two ether O atoms. A dissociative [Eu(NO_3_)_5_]^2−^ anion and two trinuclear Al^3+^ clusters balance the charge. The Eu^3+^ ion is ten-coordinated by ten O atoms from five different NO^3-^ ions, leading to a dodecahedron geometry. Through π–π interactions and van der Waals forces between two [Al_3_(*μ*_3_-O)(TEBA)_3_(H_2_O)_3_]^+^ units and electrostatic interactions among [Eu(NO_3_)_5_]^2−^ anions, a three-dimensional packing structure forms.

The ESI-MS result of compound **2** in DMF solution displays a main peak at *m*/*z* 1261.27 (Figure S10), which is consistent with the results of the mixture of compound **1** and Al^3+^ ions (*m*/*z* 1261.28, Figure S9) as well as the theoretical value of [Al_3_(*μ*_3_-O)(TEBA)_3_]^+^ (C_60_H_60_Al_3_O_25_ calcd: 1261.29). The results indicate a transformation process. Upon the addition of Al^3+^ ions to compound **1** in DMF solution, the [Na_4_Eu_2_(TEBA)_4_]^2+^ units decompose and a more stable species [Al_3_(*μ*_3_-O)(TEBA)_3_(H_2_O)_3_]^+^ forms with an increasing emission peak at 346 nm. To further explore this sensing behavior, the luminescence of the H_2_TEBA ligand and compound **2** were investigated (Figures S11 and S12). When excited at 292 nm in DMF solution, the H_2_TEBA ligand exhibits an emission peak at 340 nm, which is close to the emission peak (346 nm) of compound **2** (Figure S12) and the mixture of Al^3+^ ions and compound **1** in DMF (Figure 2). When setting the emission peak at 340 nm, the excitation spectrum of the H_2_TEBA ligand shows two peaks at 268 and 312 nm, which are different from the spectra of compound **1** at 292 nm and compound **2** at 296 nm (Figure S13). The results indicate that after being coordinated to Al^3+^ ions, the luminescence of compound **2** exhibits a slight red-shift compared with the ligand.

## 3. Materials and Methods

All reagents and solvents were commercially available and used as received without further purification. Analysis of C, H and N were carried out on an elementar vario EL elemental analyzer. The FT-IR spectra were measured with a Bruker Tensor 27 Spectrophotometer (Bruker, Karlsruhe, Germany) on KBr disks. The emission spectra in the visible region were measured on a Cary Eclipse fluorescence spectrophotometer (Agilent Technologies Inc., Santa Clara, CA, USA). The ESI-MS spectra were measured with a VG ZAB-HS spectrometer (VG, Manchester, UK). The MALDI-TOF spectra were measured on a Bruker Autoflex III TOF/TOF200 spectrometer (Bruker, Karlsruhe, Germany) using *α*-Cyano-4-hydroxycinnamic acid as matrix.

Synthesis of [Na_4_Eu_2_(TEBA)_4_(H_2_O)_4_]·[CuCl_2_]·Cl·H_2_O (**1**). A mixture of H_2_TEBA (0.3 mmol, 117.0 mg), Eu(NO_3_)_3_·6H_2_O (0.1 mmol, 44.6 mg), CuCl_2_·2H_2_O (0.2 mmol, 34.0 mg), NaOH (0.4 mmol, 16 mg) and 10 mL ethanol was sealed in 25 mL Telfon-lined stainless steel container, and heated to 160 °C for 72 h, then cooled to room-temperature (temperature decrease rate: 2 °C/h). The yellow long-stripe-like crystals were obtained in ca. 44% yield based on Eu. Elemental Analysis calcd: C, 43.67; H, 4.08%; found: C, 43.19; H, 4.19%.

Synthesis of [Al_3_(*μ*_3_-O)(TEBA)_3_(H_2_O)_3_]_2_·[Eu(NO_3_)_5_]·EtOH·0.5H_2_O (**2**). A mixture of H_2_TEBA (0.3 mmol, 117.0 mg), Eu(NO_3_)_3_·6H_2_O (0.1 mmol, 44.6 mg), AlCl_3_·6H_2_O (0.2 mmol, 48.3 mg), NaOH (0.4 mmol, 16 mg) and 10 mL ethanol was sealed in 25 mL Telfon-lined stainless steel container, and heated to 160 °C for 72 h, then cooled to room-temperature (temperature decrease rate: 2 °C/h). The colorless long-stripe-like crystals were obtained in ca. 32% yield based on Al. Elemental Analysis calcd: C, 46.53; H, 4.45%; found: C, 45.94; H, 4.16%.

Suitable crystals of compound **1** and **2** were selected and mounted on a SuperNova, (Single source at offset) Eos diffractometer equipped with graphite monochromated Mo *K*α radiation (*λ* = 0.71073 Å) under the temperature 120(2) K. Using Olex2 programme [45], the structures were solved with the ShelXS structure solution program using Direct Methods and refined with the ShelXL refinement package using Least Squares minimisation [46]. Both structures were treated as twinning crystal.

Crystallographic data for **1** and **2** reported in this paper have been deposited with the Cambridge Crystallographic Data Centre as supplementary publication no. CCDC 2215277 and 2215275, respectively. This data can be obtained free of charge from the Cambridge Crystallographic Data Centre via www.ccdc.cam.ac.uk/data_request/cif (accessed on 26 October 2022).

## 4. Conclusions

In conclusion, a novel luminescent dinuclear europium compound with a crown ether analogous ligand was synthesized through solvent–thermal reaction and was structurally characterized. The luminescent investigations indicate that this compound is a promising luminescent probe for Al^3+^ ions. Through transformation, a new, more stable trinuclear aluminum compound was formed. The luminescent intensity of the characteristic emissions of Eu^3+^ decreased, and a new emission peak appeared at 346 nm and increased rapidly as the concentration of Al^3+^ increased. This transformation mechanism provided a novel OFF–ON and ON–OFF luminescent probe, which improved the sensitivity of this sensor. We believe that this novel probe will open a new route to the design of lanthanide luminescent probes.

## Data Availability

Data is contained within the article or supplementary material.

## Supplementary Materials

The following supporting information can be downloaded at: https://www.mdpi.com/article/10.3390/molecules27248761/s1, Figure S1: ORTEP view of the X-ray crystal structure of compound **1**. Figure S2: ORTEP view of the X-ray crystal structure of compound **2**. Table S1: Crystal data and structure refinement for **1** and **2**. Table S2: Bond lengths for **1**. Table S3: Bond lengths for **2**. Figure S3: Luminescence decay kinetics of the Eu^3+^ emission (618 nm) in compound **1** under 292 nm excitation at room temperature. The green line is the fit for delay time. Figure S4: The luminescent emission spectra (excited at 292 nm) of compound **1** in DMF upon the addition up to 2 equiv of Ca^2+^, Cd^2+^, Co^2+^, Cu^2+^, Fe^3+^, K^+^, Mg^2+^, Ni^2+^ and Pb^2+^ ions. Figure S5: The relationship between the emission intensity at 346 nm and the equivalent addition of Al^3+^ ions. Figure S6: The emission spectra (excited at 292 nm) of compound **1** in DMF at room temperature in the presence of different low concentration of Al^3+^ ions. It reveals that the detection limit of **1** for sensing Al^3+^ ions is about 5 × 10^−6^ M. Figure S7: The ESI-MS of compound **1** in DMF solution. Figure S8: The MALDI-TOF of compound **1**. Figure S9: The ESI-MS of compound **1** in DMF solution after addition of Al^3+^ ions. Figure S10: The ESI-MS of compound **2** in DMF solution. Figure S11: The luminescent emission spectra of ligand H_2_TEBA in DMF solution with an emission peak at 340 nm. Figure S12: The luminescent emission spectra of compound **2** in DMF solution with a main emission peak at 346 nm. Figure S13: The excitation spectra of ligand H_2_TEBA, compound **1** and compound **2** in DMF solution observed at their highest emission peak, respectively.

## Sample Availability

Samples of the compounds **1** and **2** are available from the authors.
