# Supplementary material for: Dinuclear Lanthanide Compound as a Promising Luminescent Probe for Al3+ Ions
Source: Molecules. 2022 Dec 10;27(24):8761. doi: 10.3390/molecules27248761 (PMC9783527; doi:10.3390/molecules27248761)
Supplement: Supplementary file 1 [file molecules-27-08761-s001.zip › molecules-2030348-supplementary.pdf]

Supporting Information

Dinuclear Lanthanide Compound as a Promising Luminescent Probe for Al^{3+} Ions

Zhi Chen[1,*], Yinghao Xie[1], Zhanbo Li[2] and Tao Lin[2,*]

[1]College of Chemistry and Environmental Engineering, Shenzhen University, Shenzhen, 518071, China

[2]College of New Materials and New Energies, Shenzhen Technology University, Shenzhen 518118, China

* Correspondence: zhi.chen@szu.edu.cn (Z.C.); lintao@sztu.edu.cn (T.L.)

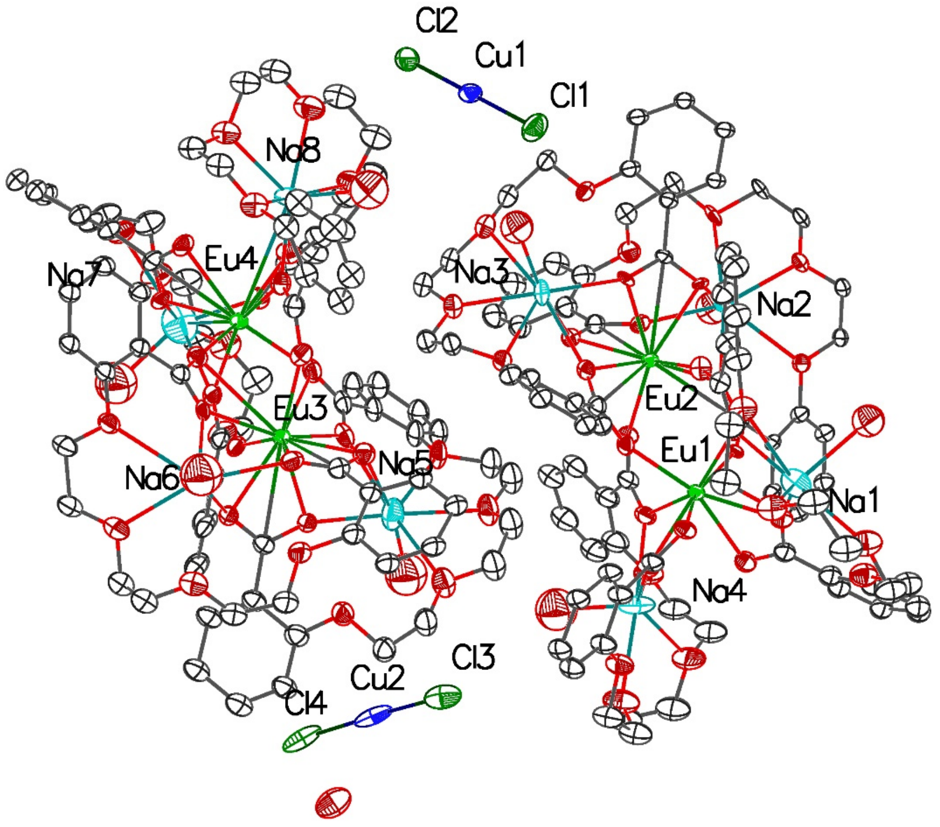

Figure S1. ORTEP view of the X-ray crystal structure of compound **1**. Atoms are shown as 30% thermal ellipsoids. Hydrogen atoms are omitted for clarity.

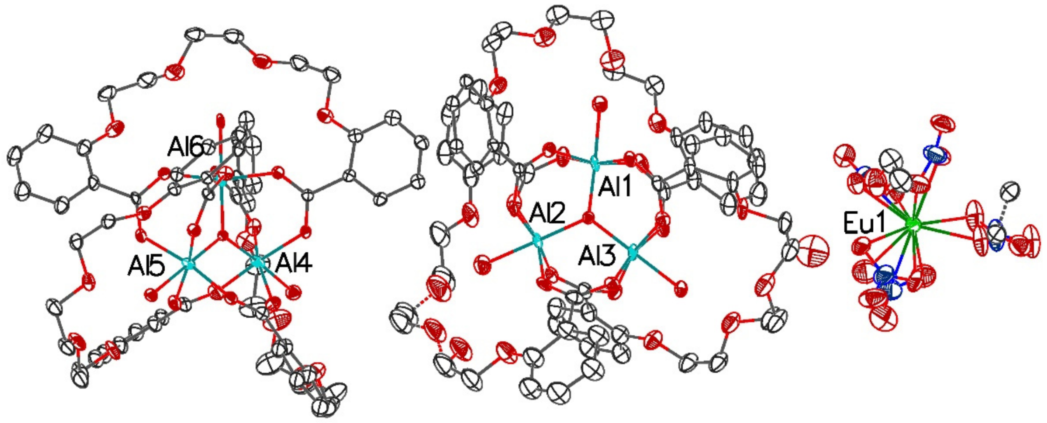

Figure S2. ORTEP view of the X-ray crystal structure of compound **2**. Atoms are shown as 30% thermal ellipsoids. Hydrogen atoms are omitted for clarity.

Table S1. Crystal data and structure refinement for **1** and **2**.

Identification code	**1**	**2**
Empirical formula	$C_{80}H_{89}Cl_3CuEu_2Na_4O_{36.5}$	$C_{122}H_{139}Al_6EuN_5O_{72.5}$
Formula weight	2200.28	3149.21
Temperature/K	120(2)	120(2)
Crystal system	triclinic	triclinic
Space group	$P\bar{1}$	$P\bar{1}$
a/Å	16.0944(8)	10.8250(6)
b/Å	22.3421(10)	20.320(3)
c/Å	26.5207(15)	32.429(3)
α/°	93.324(4)	90.834(8)
β/°	105.705(5)	90.347(6)
γ/°	98.828(4)	102.008(8)
Volume/Å^3	9021.8(8)	6976.1(13)
Z	4	2
ρ_{calc} g/cm^3	1.620	1.499
μ/mm^{-1}	1.802	0.594
F(000)	4444.0	3254.0
Crystal size/mm^3	0.4 × 0.1 × 0.1	0.4 × 0.1 × 0.1
Radiation	Mo Kα (λ = 0.71073)	Mo Kα (λ = 0.71073)
2Θ range for data collection/°	4.72 to 50.02	4.696 to 50.02
Index ranges	$-19 \le h \le 19$, $-26 \le k \le 26$, $-31 \le l \le 31$	$-12 \le h \le 12$, $-24 \le k \le 23$, $-38 \le l \le 38$
Reflections collected	31739	23948
Independent reflections	31739 [R_{sigma} = 0.2351]	23948 [R_{sigma} = 0.2377]
Data/restraints/parameters	31739/1483/2302	23948/1414/1943
Goodness-of-fit on F^2	1.189	0.998

Final R indexes [$I >= 2\sigma (I)$]	$R_1 = 0.1370$, $wR_2 = 0.3610$	$R_1 = 0.1013$, $wR_2 = 0.2092$
Final R indexes [all data]	$R_1 = 0.2189$, $wR_2 = 0.3935$	$R_1 = 0.1678$, $wR_2 = 0.2336$
Largest diff. peak/hole / e Å^{-3}	6.05/-2.80	1.24/-0.92

Table S2. Bond lengths for **1**.

Atom	Atom	Length/Å	Atom	Atom	Length/Å
Eu1	O1	2.314(14)	C44	C45	1.40(4)
Eu1	O16	2.332(18)	C45	C46	1.31(4)
Eu1	O18	2.397(15)	C46	C47	1.49(4)
Eu1	O23	2.440(18)	C47	O19	1.35(3)
Eu1	O24	2.476(19)	C48	C49	1.49(4)
Eu1	O25	2.410(16)	C48	O19	1.47(3)
Eu1	O31	2.480(16)	C49	O20	1.41(4)
Eu1	O32	2.438(15)	C50	C51	1.44(4)
Eu2	O2	2.349(15)	C50	O20	1.43(3)
Eu2	O7	2.449(16)	C51	O21	1.57(4)
Eu2	O8	2.488(15)	C52	C53	1.51(4)
Eu2	O9	2.385(17)	C52	O21	1.42(4)
Eu2	O10	2.458(15)	C53	O22	1.47(3)
Eu2	O15	2.402(15)	C54	C55	1.41(4)
Eu2	O17	2.319(16)	C54	C59	1.44(4)
Eu2	O26	2.265(16)	C54	O22	1.37(4)
Eu3	O37	2.502(18)	C55	C56	1.27(4)
Eu3	O38	2.476(18)	C56	C57	1.36(4)
Eu3	O43	2.421(16)	C57	C58	1.38(4)
Eu3	O44	3.123(18)	C58	C59	1.38(4)
Eu3	O45	2.316(18)	C59	C60	1.43(4)
Eu3	O60	2.336(18)	C60	O23	1.26(3)
Eu3	O61	2.522(15)	C60	O24	1.26(3)
Eu3	O62	2.370(17)	C61	C62	1.536(18)
Eu3	O67	2.365(18)	C61	O25	1.35(3)
Eu3	O68	3.140(17)	C61	O26	1.22(3)
Eu4	O44	2.317(17)	C62	C67	1.36(4)
Eu4	O46	2.381(17)	C64	C65	1.40(4)
Eu4	O51	2.399(16)	C65	C66	1.35(4)
Eu4	O52	2.501(17)	C66	C67	1.39(4)
Eu4	O53	2.564(19)	C67	O27	1.39(4)
Eu4	O54	2.453(16)	C68	C69	1.53(4)

Table S2. Bond lengths for **1**.

Atom	Atom	Length/Å	Atom	Atom	Length/Å
Eu4	O59	2.447(19)	C68	O27	1.50(4)
Eu4	O68	2.37(2)	C69	O28	1.41(4)
Cu1	Cl1	2.104(9)	C70	C71	1.44(5)
Cu1	Cl2	2.071(9)	C70	O28	1.44(4)
Cu2	Cl3	2.087(11)	C71	O29	1.24(4)
Cu2	Cl4	2.124(12)	C72	C73	1.53(4)
Na1	O18	2.45(2)	C72	O29	1.44(4)
Na1	O19	2.75(2)	C73	O30	1.44(3)
Na1	O20	2.44(2)	C74	C75	1.37(4)
Na1	O21	2.80(2)	C74	C79	1.42(4)
Na1	O23	2.30(2)	C74	O30	1.33(3)
Na1	O34	2.49(2)	C75	C76	1.39(4)
Na2	O2	2.482(16)	C76	C77	1.40(4)
Na2	O3	2.66(2)	C77	C78	1.39(3)
Na2	O4	2.419(18)	C78	C79	1.35(4)
Na2	O5	2.474(19)	C79	C80	1.55(3)
Na2	O7	2.371(19)	C80	O31	1.30(3)
Na2	O10	2.63(2)	C80	O32	1.23(3)
Na2	O36	2.31(3)	C81	C82	1.524(18)
Na3	O8	2.77(2)	C81	O37	1.29(3)
Na3	O9	2.336(19)	C81	O38	1.24(3)
Na3	O12	2.80(2)	C82	C83	1.35(4)
Na3	O13	2.38(2)	C82	C87	1.37(3)
Na3	O14	2.79(2)	C83	C84	1.40(4)
Na3	O15	2.41(2)	C84	C85	1.40(4)
Na3	O35	2.30(4)	C85	C86	1.26(4)
Na4	O25	2.445(19)	C86	C87	1.42(4)
Na4	O27	2.74(3)	C87	O39	1.48(3)
Na4	O28	2.46(3)	C88	C89	1.49(4)
Na4	O29	2.62(2)	C88	O39	1.37(3)
Na4	O32	2.31(2)	C89	O40	1.52(4)
Na4	O33	2.20(4)	C90	C91	1.52(4)
Na5	O37	2.71(2)	C90	O40	1.37(3)
Na5	O62	2.34(2)	C91	O41	1.44(3)
Na5	O64	2.72(2)	C92	C93	1.61(4)
Na5	O65	2.52(2)	C92	O41	1.45(3)
Na5	O66	2.83(2)	C93	O42	1.39(3)
Na5	O67	2.58(2)	C94	C95	1.37(4)

Table S2. Bond lengths for **1**.

Atom	Atom	Length/Å	Atom	Atom	Length/Å
Na5	O70	2.28(4)	C94	C99	1.39(4)
Na6	O38	2.48(2)	C94	O42	1.37(3)
Na6	O41	2.47(2)	C95	C96	1.34(4)
Na6	O42	2.77(2)	C96	C97	1.37(4)
Na6	O43	2.40(2)	C97	C98	1.35(4)
Na6	O61	2.50(2)	C98	C99	1.44(4)
Na6	O69	2.36(3)	C99	C100	1.48(4)
Na7	O52	2.43(2)	C100	O43	1.24(3)
Na7	O54	2.23(3)	C100	O44	1.26(3)
Na7	O55	2.98(3)	C101	C102	1.50(4)
Na7	O56	2.60(3)	C101	O45	1.28(3)
Na7	O57	2.45(3)	C101	O46	1.29(3)
Na7	O59	2.95(3)	C102	C103	1.48(4)
Na7	O71	2.42(4)	C102	C107	1.37(4)
Na8	O46	2.45(2)	C103	C104	1.44(4)
Na8	O47	2.62(2)	C104	C105	1.30(4)
Na8	O48	2.47(3)	C105	C106	1.40(4)
Na8	O49	2.55(2)	C106	C107	1.43(4)
Na8	O50	2.96(2)	C107	O47	1.39(3)
Na8	O51	2.37(2)	C108	C109	1.50(4)
Na8	O72	2.37(3)	C108	O47	1.44(4)
C1	C2	1.36(3)	C109	O48	1.46(4)
C1	C6	1.38(3)	C110	C111	1.51(4)
C1	C7	1.50(3)	C110	O48	1.43(3)
C2	C3	1.45(4)	C111	O49	1.34(4)
C3	C4	1.32(3)	C112	C113	1.504(19)
C4	C5	1.37(3)	C112	O49	1.428(18)
C4AA	C62	1.33(4)	C113	O50	1.42(3)
C4AA	C64	1.43(4)	C114	C115	1.36(4)
C5	C6	1.31(3)	C114	C119	1.35(4)
C6	O3	1.43(3)	C114	O50	1.43(4)
C7	O1	1.33(3)	C115	C116	1.39(4)
C7	O2	1.22(3)	C116	C117	1.30(4)
C8	C9	1.58(3)	C117	C118	1.42(4)
C8	O3	1.44(3)	C118	C119	1.38(4)
C9	O4	1.37(3)	C119	C120	1.48(4)
C10	C11	1.42(4)	C120	O51	1.22(3)
C10	O4	1.39(3)	C120	O52	1.25(3)

Table S2. Bond lengths for **1**.

Atom	Atom	Length/Å	Atom	Atom	Length/Å
C11	O5	1.48(3)	C121	C122	1.47(4)
C12	C13	1.56(3)	C121	O53	1.26(3)
C12	O5	1.47(3)	C121	O54	1.33(3)
C13	O6	1.43(3)	C122	C123	1.39(4)
C14	C15	1.42(4)	C122	C127	1.37(4)
C14	C19	1.35(4)	C123	C124	1.36(4)
C14	O6	1.44(3)	C124	C125	1.42(4)
C15	C16	1.34(4)	C125	C126	1.34(4)
C16	C17	1.36(4)	C126	C127	1.45(4)
C17	C18	1.38(4)	C127	O55	1.39(4)
C18	C19	1.40(3)	C128	C129	1.45(4)
C19	C20	1.53(3)	C128	O55	1.48(3)
C20	O7	1.22(3)	C129	O56	1.50(4)
C20	O8	1.32(3)	C130	C131	1.61(4)
C21	C22	1.52(3)	C130	O56	1.38(4)
C21	O9	1.24(3)	C131	O57	1.41(3)
C21	O10	1.28(3)	C132	C133	1.48(4)
C22	C23	1.40(3)	C132	O57	1.31(4)
C22	C27	1.36(4)	C133	O58	1.50(4)
C23	C24	1.38(3)	C134	C135	1.39(4)
C24	C25	1.34(3)	C134	C139	1.40(4)
C25	C26	1.36(3)	C134	O58	1.35(3)
C26	C27	1.39(3)	C135	C136	1.35(4)
C27	O11	1.39(3)	C136	C137	1.32(4)
C28	C29	1.61(4)	C137	C138	1.42(4)
C28	O11	1.42(3)	C138	C139	1.45(4)
C29	O12	1.44(3)	C139	C140	1.44(4)
C30	C31	1.44(4)	C140	O59	1.29(3)
C30	O12	1.45(3)	C140	O60	1.26(3)
C31	O13	1.44(3)	C141	C142	1.50(4)
C32	C33	1.47(4)	C141	O61	1.24(3)
C32	O13	1.42(3)	C141	O62	1.35(3)
C33	O14	1.42(3)	C142	C143	1.28(4)
C34	C35	1.50(4)	C142	C147	1.48(4)
C34	C39	1.37(4)	C143	C144	1.44(4)
C34	O14	1.36(3)	C144	C145	1.46(4)
C35	C36	1.39(4)	C145	C146	1.30(4)
C36	C37	1.39(4)	C146	C147	1.39(4)

Table S2. Bond lengths for **1**.

Atom	Atom	Length/Å	Atom	Atom	Length/Å
C37	C38	1.34(4)	C147	O63	1.38(3)
C38	C39	1.45(4)	C148	C149	1.468(18)
C39	C40	1.46(4)	C148	O63	1.45(3)
C40	O15	1.29(3)	C149	O64	1.45(3)
C40	O16	1.30(3)	C150	C151	1.38(4)
C41	C42	1.48(4)	C150	O64	1.43(3)
C41	O17	1.25(3)	C151	O65	1.36(3)
C41	O18	1.32(3)	C152	C153	1.44(4)
C42	C43	1.35(4)	C152	O65	1.34(3)
C42	C47	1.39(4)	C153	O66	1.44(3)
C43	C44	1.39(4)	C154	C155	1.36(4)
C158	C159	1.38(4)	C154	C159	1.31(4)
C159	C160	1.55(4)	C154	O66	1.38(3)
C160	O67	1.25(3)	C155	C156	1.33(4)
C160	O68	1.18(3)	C156	C157	1.35(4)
Cl10	O33[1]	2.07(7)	C157	C158	1.44(4)

[1]2-X,1-Y,2-Z

Table S3. Bond lengths for **2**.

Atom	Atom	Length/Å	Atom	Atom	Length/Å
Eu1	O57	2.488(11)	C48	C49	1.47(2)
Eu1	O59	2.450(12)	C48	O19	1.431(18)
Eu1	O61	2.408(11)	C49	O20	1.453(16)
Eu1	O62	2.434(11)	C50	C51	1.49(2)
Eu1	O63	2.484(11)	C50	O20	1.396(19)
Eu1	O64	2.416(13)	C51	O21	1.445(18)
Eu1	O67	2.397(11)	C52	C53	1.450(19)
Eu1	O68	2.522(12)	C52	O21	1.451(17)
Eu1	O69	2.398(13)	C53	O22	1.387(17)
Eu1	O71	2.454(13)	C54	C55	1.479(18)
Al1	O2	1.945(9)	C54	C59	1.360(19)
Al1	O16	1.943(9)	C54	O22	1.301(16)
Al1	O18	1.878(10)	C55	C56	1.333(18)
Al1	O23	1.861(10)	C56	C57	1.351(19)
Al1	O25	1.780(11)	C57	C58	1.377(18)
Al1	O26	1.946(11)	C58	C59	1.410(18)
Al2	O1	1.885(11)	C59	C60	1.483(19)
Al2	O7	1.877(11)	C60	O23	1.301(16)

Table S3. Bond lengths for **2**.

Atom	Atom	Length/Å	Atom	Atom	Length/Å
Al2	O9	1.921(11)	C60	O24	1.263(17)
Al2	O24	1.927(10)	C61	C62	1.526(18)
Al2	O25	1.821(9)	C61	O29	1.228(16)
Al2	O27	1.969(11)	C61	O30	1.242(15)
Al3	O8	1.921(11)	C62	C63	1.381(17)
Al3	O10	1.884(10)	C62	C67	1.346(19)
Al3	O15	1.881(9)	C63	C64	1.398(17)
Al3	O17	1.936(10)	C64	C65	1.302(19)
Al3	O25	1.840(10)	C65	C66	1.368(19)
Al3	O28	1.965(10)	C66	C67	1.396(18)
Al4	O29	1.939(9)	C67	O31	1.380(16)
Al4	O44	1.910(10)	C68	C69	1.456(18)
Al4	O46	1.876(11)	C68	O31	1.429(16)
Al4	O51	1.868(10)	C69	O32	1.414(17)
Al4	O53	1.797(9)	C70	C71	1.50(2)
Al4	O56	2.002(10)	C70	O32	1.443(16)
Al5	O36	1.893(10)	C71	O33	1.427(17)
Al5	O38	1.862(9)	C72	C73	1.528(19)
Al5	O43	1.863(9)	C72	O33	1.382(15)
Al5	O45	1.913(10)	C73	O34	1.412(17)
Al5	O53	1.812(9)	C74	C75	1.39(2)
Al5	O55	1.969(10)	C74	C79	1.40(2)
Al6	O30	1.887(9)	C74	O34	1.330(17)
Al6	O35	1.887(10)	C75	C76	1.40(2)
Al6	O37	1.916(9)	C76	C77	1.36(2)
Al6	O52	1.944(9)	C77	C78	1.374(19)
Al6	O53	1.834(11)	C78	C79	1.380(19)
Al6	O54	1.927(10)	C79	C80	1.489(18)
C1	C2	1.49(2)	C80	O35	1.245(15)
C1	O1	1.225(18)	C80	O36	1.268(17)
C1	O2	1.314(17)	C81	C82	1.510(17)
C2	C3	1.43(2)	C81	O37	1.248(15)
C2	C7	1.34(2)	C81	O38	1.262(15)
C3	C4	1.41(2)	C82	C83	1.395(18)
C3	O3	1.33(2)	C82	C87	1.404(18)
C4	C5	1.32(2)	C83	C84	1.386(17)
C5	C6	1.35(2)	C84	C85	1.418(18)
C6	C7	1.45(2)	C85	C86	1.354(18)

Table S3. Bond lengths for **2**.

Atom	Atom	Length/Å	Atom	Atom	Length/Å
C8	C9	1.53(3)	C86	C87	1.414(16)
C8	O3	1.392(17)	C87	O39	1.329(15)
C9	O4	1.33(2)	C88	C89	1.429(17)
C10	C11	1.33(5)	C88	O39	1.432(15)
C10	O4	1.55(4)	C89	O40	1.406(16)
C10'	C11'	1.35(5)	C90	C91	1.45(2)
C10'	O4	1.63(4)	C90	O40	1.415(15)
C11	O5	1.53(4)	C91	O41	1.451(16)
C11'	O5'	1.37(4)	C92	C93	1.515(19)
C12	C13	1.42(2)	C92	O41	1.359(18)
C12	O5	1.338(17)	C93	O42	1.360(16)
C12	O5'	1.20(3)	C94	C95	1.406(18)
C13	O6	1.431(18)	C94	C99	1.41(2)
C14	C15	1.50(2)	C94	O42	1.302(16)
C14	O7	1.255(16)	C95	C96	1.336(19)
C14	O8	1.294(16)	C96	C97	1.37(2)
C15	C16	1.39(2)	C97	C98	1.404(18)
C15	C20	1.41(2)	C98	C99	1.395(19)
C16	C17	1.34(2)	C99	C100	1.508(19)
C16	O6	1.365(18)	C100	O43	1.259(15)
C17	C18	1.36(2)	C100	O44	1.231(16)
C18	C19	1.30(2)	C101	C102	1.54(2)
C19	C20	1.47(2)	C101	O45	1.278(16)
C21	C22	1.53(2)	C101	O46	1.245(16)
C21	O9	1.219(17)	C102	C103	1.398(19)
C21	O10	1.227(16)	C102	C107	1.33(2)
C22	C23	1.400(19)	C103	C104	1.37(2)
C22	C27	1.37(2)	C104	C105	1.33(2)
C23	C24	1.42(2)	C105	C106	1.37(2)
C24	C25	1.36(2)	C106	C107	1.43(2)
C25	C26	1.375(19)	C107	O47	1.377(18)
C26	C27	1.357(19)	C108	C109	1.47(2)
C27	O11	1.431(17)	C108	O47	1.428(18)
C28	C29	1.511(18)	C109	O48	1.311(18)
C28	O11	1.474(16)	C110	C111	1.49(2)
C29	O12	1.384(19)	C110	O48	1.443(19)
C30	C31	1.431(15)	C111	O49	1.50(2)
C30	O12	1.382(16)	C112	C113	1.49(2)

Table S3. Bond lengths for **2**.

Atom	Atom	Length/Å	Atom	Atom	Length/Å
C31	O13	1.424(19)	C112	O49	1.368(18)
C32	C33	1.52(2)	C113	O50	1.474(16)
C32	O13	1.415(19)	C114	C115	1.401(19)
C33	O14	1.434(16)	C114	C119	1.408(19)
C34	C35	1.372(18)	C114	O50	1.321(18)
C34	C39	1.446(19)	C115	C116	1.32(2)
C34	O14	1.300(16)	C116	C117	1.359(19)
C35	C36	1.36(2)	C117	C118	1.398(18)
C36	C37	1.344(19)	C118	C119	1.38(2)
C37	C38	1.412(17)	C119	C120	1.464(19)
C38	C39	1.390(19)	C120	O51	1.249(17)
C39	C40	1.525(18)	C120	O52	1.271(16)
C40	O15	1.239(16)	C121	C122	1.49(4)
C40	O16	1.221(17)	C122	O72	1.33(3)
C41	C42	1.46(2)	C123	C124	1.16(4)
C41	O17	1.265(19)	C124	O74	1.29(4)
C41	O18	1.256(18)	N1	O57	1.262(17)
C42	C43	1.42(2)	N1	O58	1.200(17)
C42	C47	1.39(2)	N1	O59	1.248(18)
C43	C44	1.31(2)	N2	O60	1.243(16)
C44	C45	1.40(2)	N2	O61	1.299(16)
C45	C46	1.39(2)	N2	O62	1.224(16)
C46	C47	1.34(2)	N3	O63	1.327(18)
C47	O19	1.373(18)	N3	O64	1.237(17)
N5	O70	1.332(19)	N3	O65	1.239(18)
N5	O71	1.218(19)	N4	O66	1.224(17)
N4	O68	1.303(19)	N4	O67	1.271(18)
N5	O69	1.215(18)			

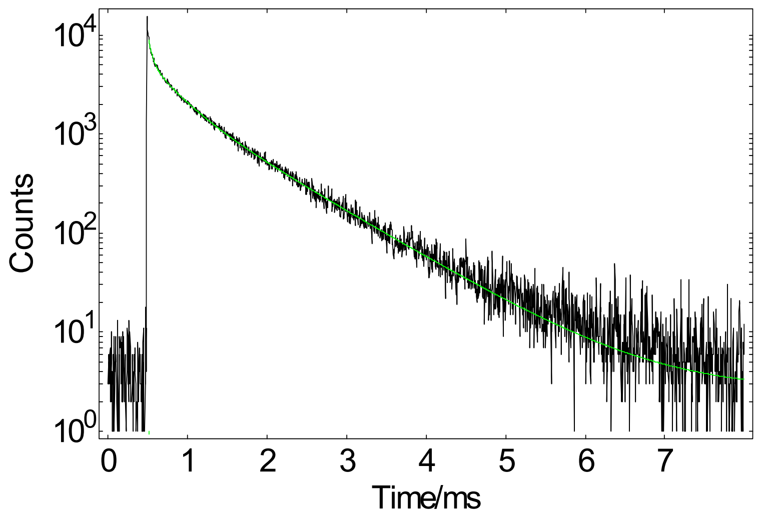

Figure S3. Luminescence decay kinetics of the Eu^{3+} emission (618 nm) in compound **1** under 292 nm excitation at room temperature. The green line is the fit for delay time.

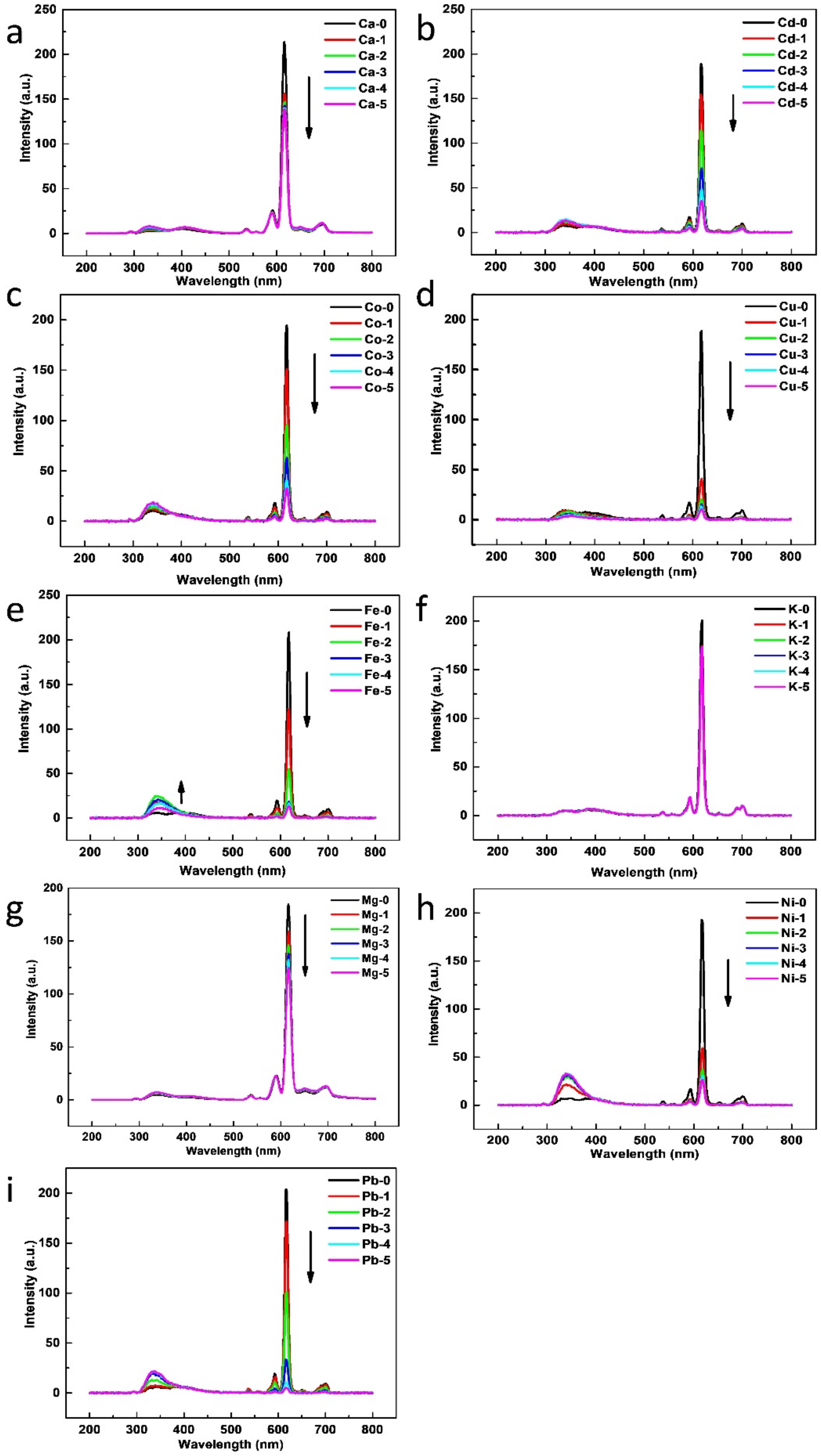

Figure S4. a)-i) The luminescent emission spectra (excited at 292 nm) of compound **1** in DMF upon the addition up to 2 equiv of Ca^{2+}, Cd^{2+}, Co^{2+}, Cu^{2+}, Fe^{3+}, K^{+}, Mg^{2+}, Ni^{2+} and Pb^{2+} ions.

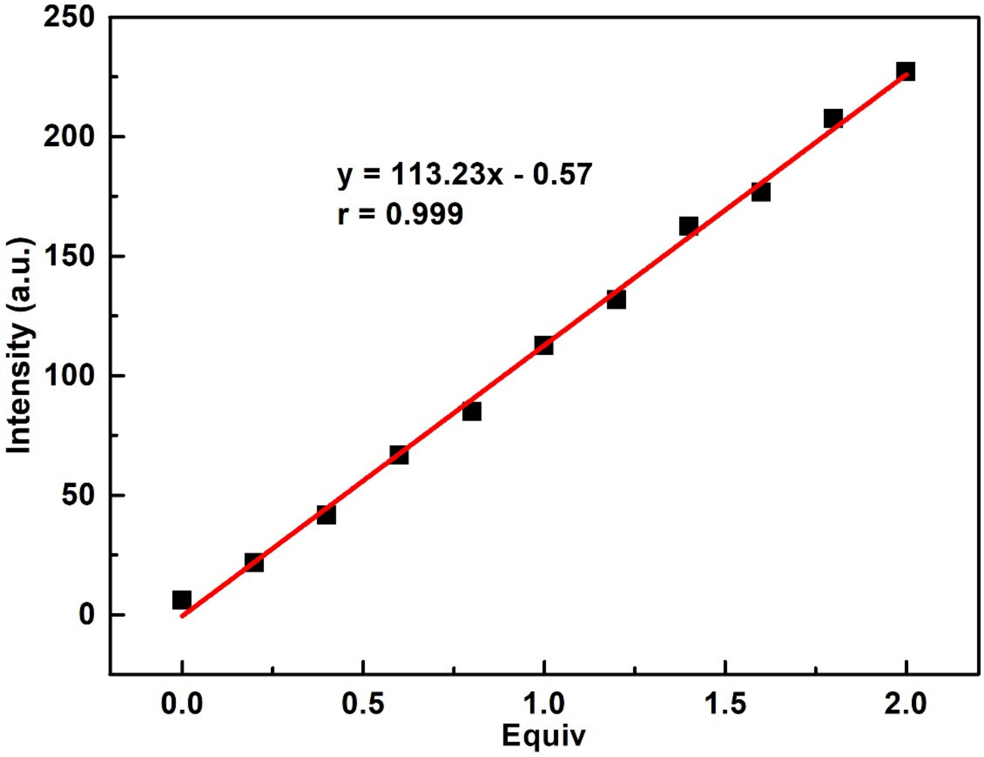

Figure S5. The relationship between the emission intensity at 346 nm and the equivalent addition of Al^{3+} ions. The red line indicates the linear fit.

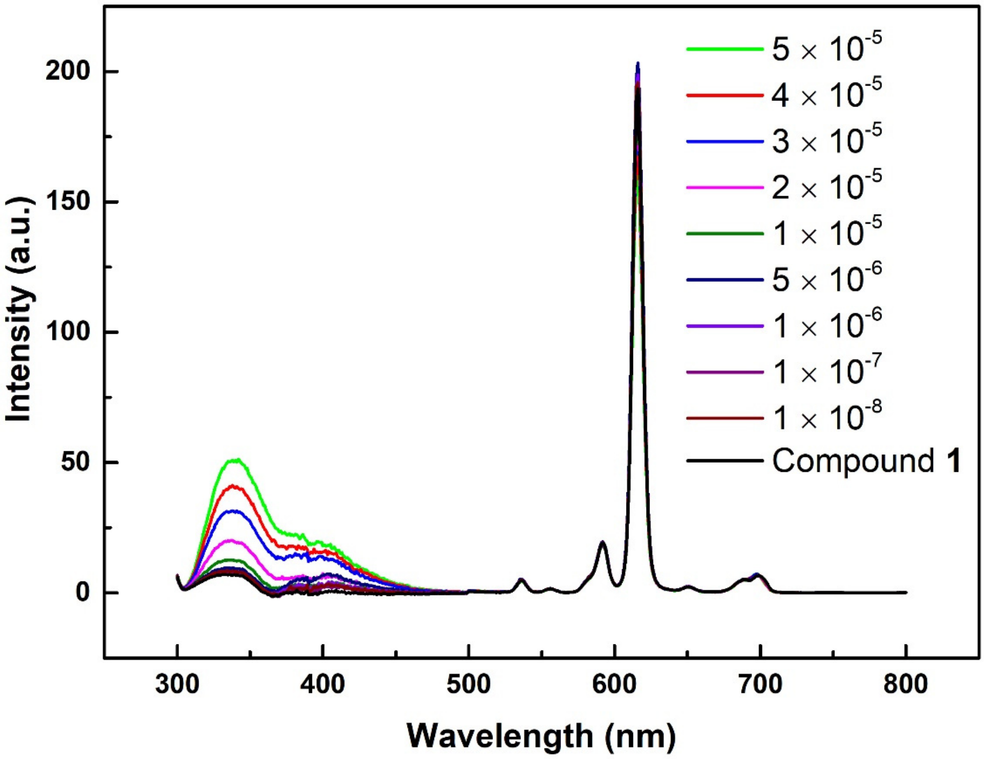

Figure S6. The emission spectra (excited at 292 nm) of compound **1** in DMF at room temperature in the presence of different low concentrations of Al^{3+} ions. It reveals that the detection limit of **1** for sensing Al^{3+} ions is about 5×10^{-6} M.

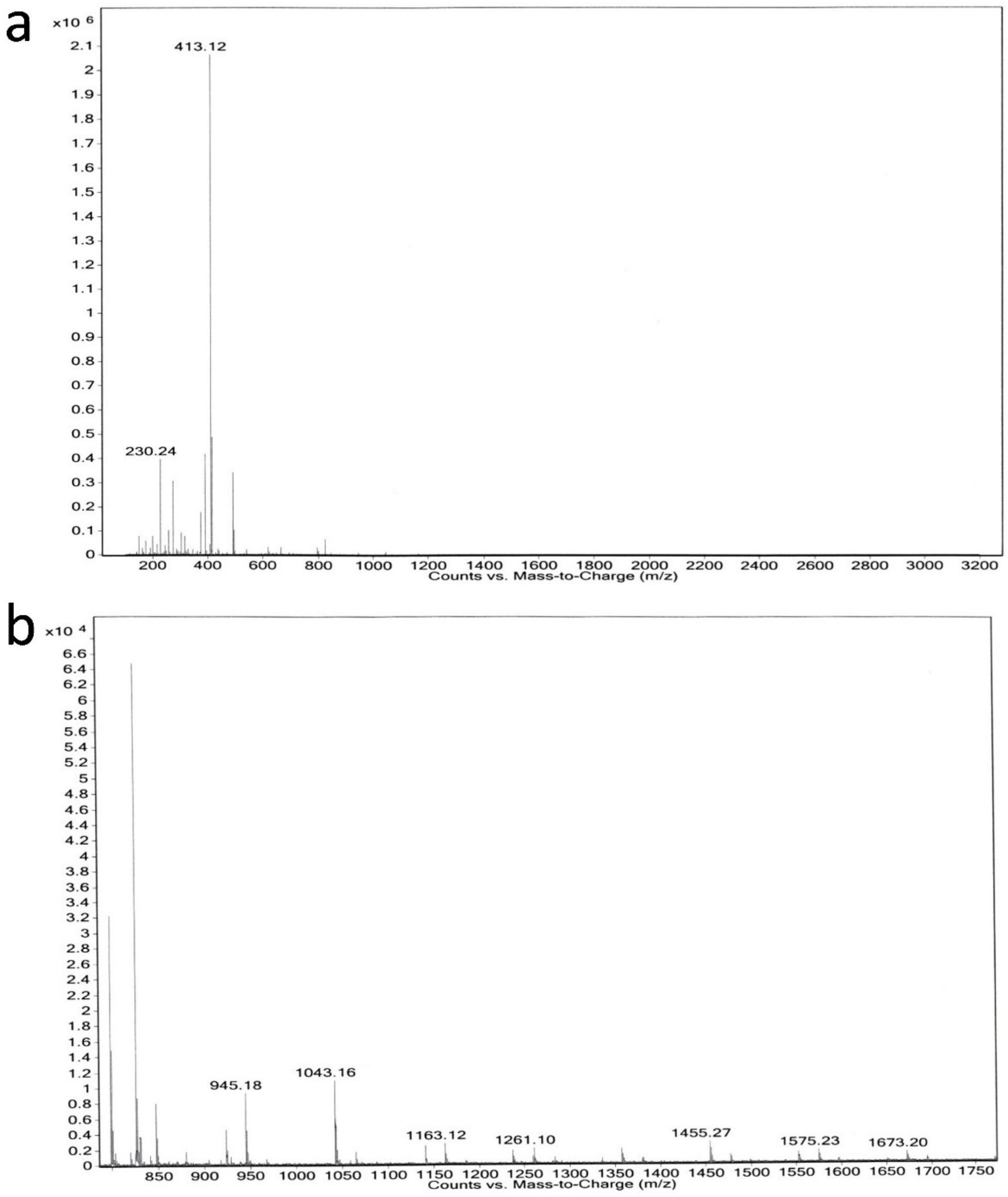

Figure S7. The ESI-MS of compound **1** in DMF solution. a) Full-range spectrum, b) Close-up spectrum from 800 to 1800.

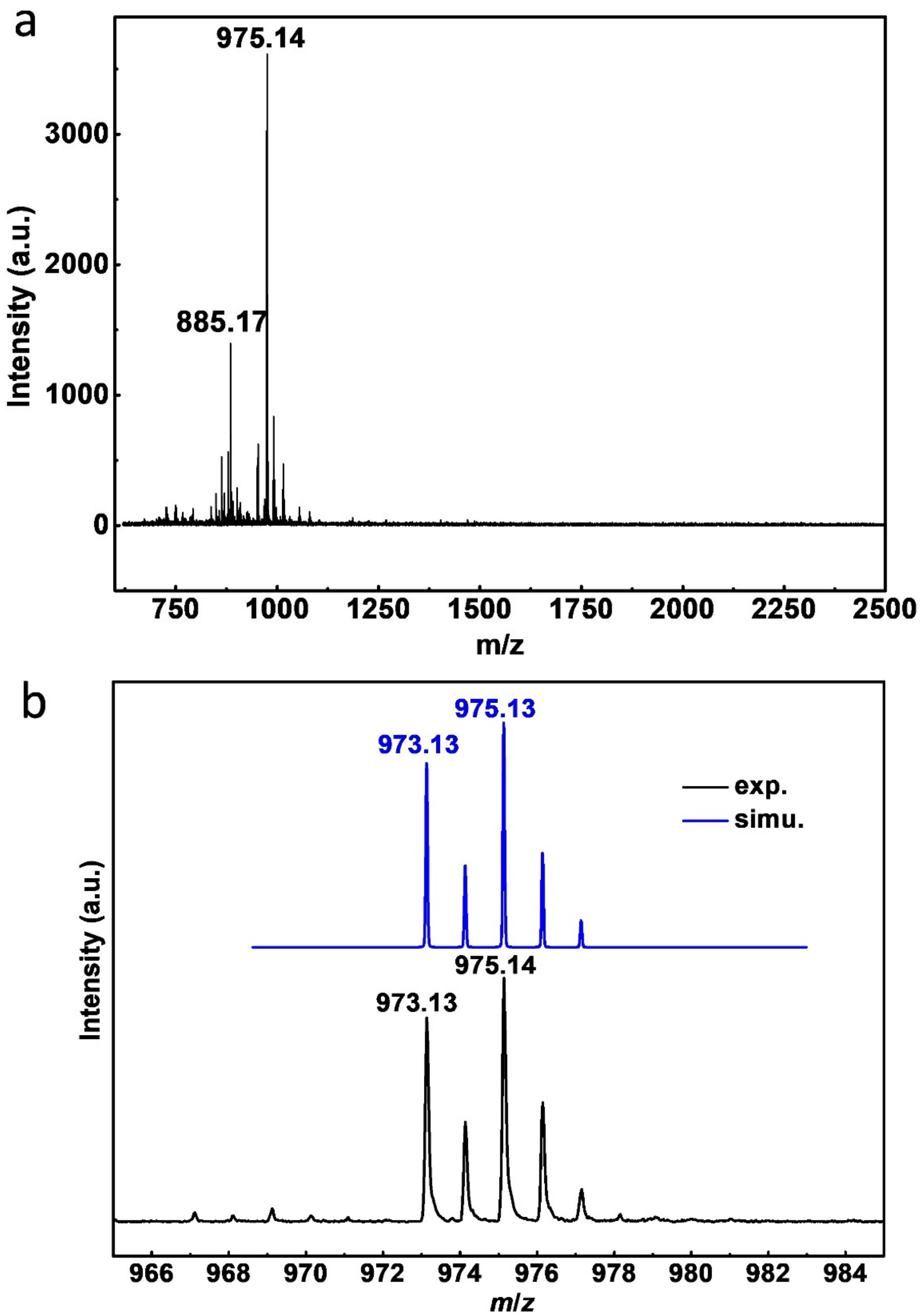

Figure S8. The MALDI-TOF of compound **1**.

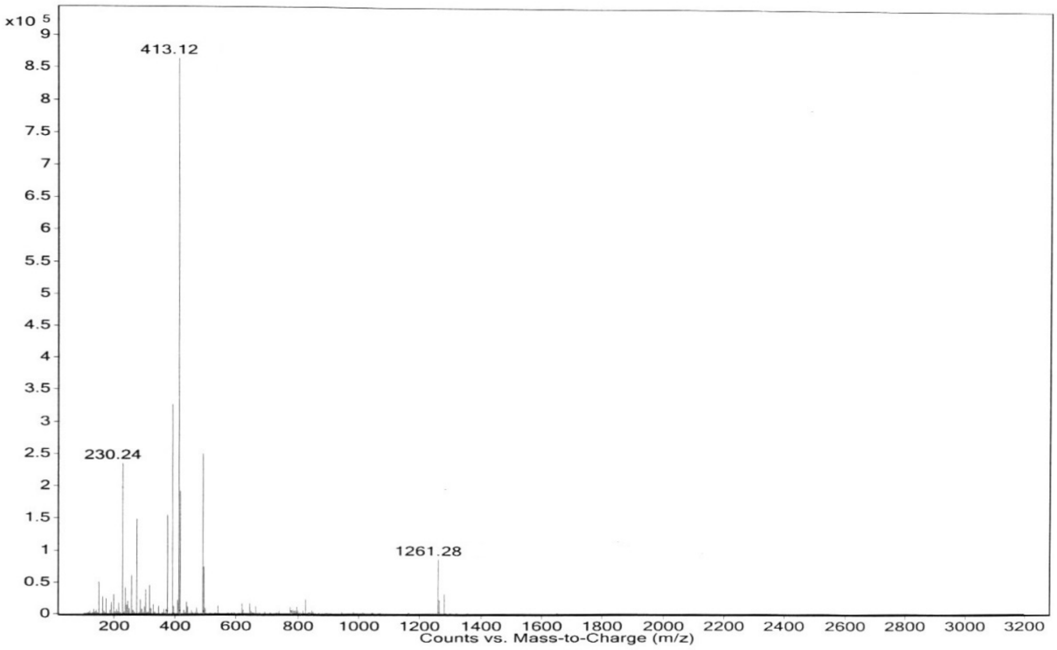

Figure S9. The ESI-MS of compound **1** in DMF solution after addition of Al^{3+} ions.

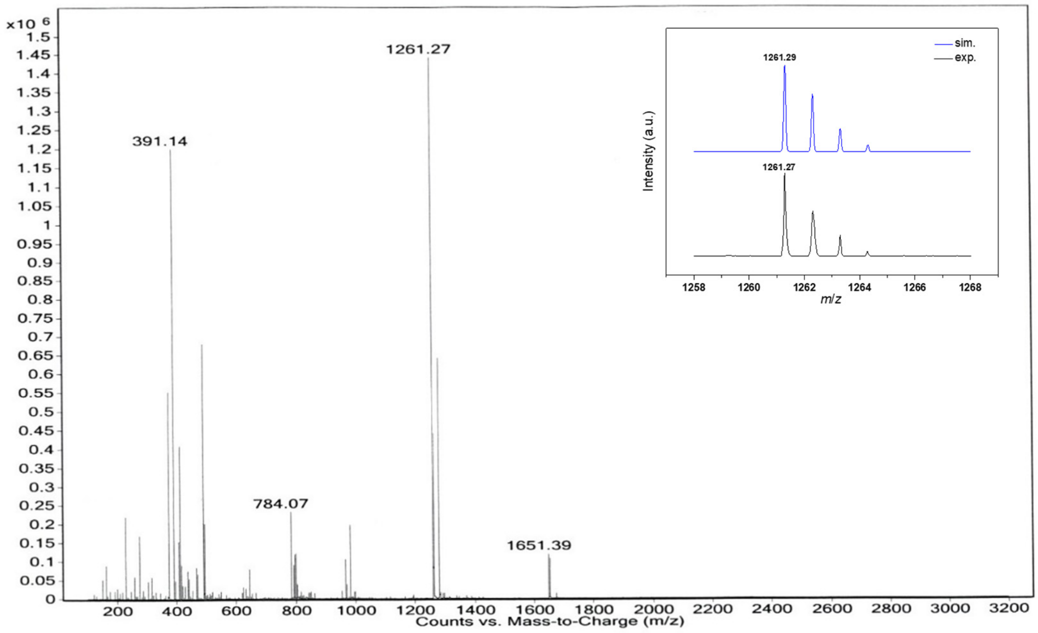

Figure S10. The ESI-MS of compound **2** in DMF solution.

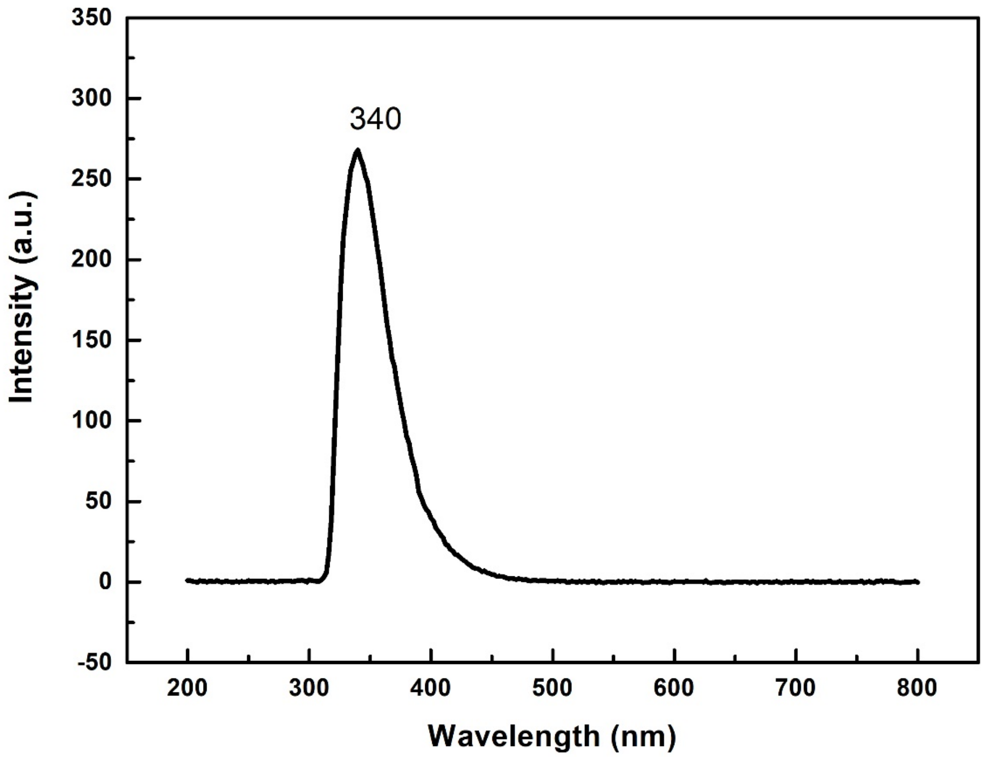

Figure S11. The luminescent emission spectra of ligand H_2TEBA in DMF solution with an emission peak at 340 nm.

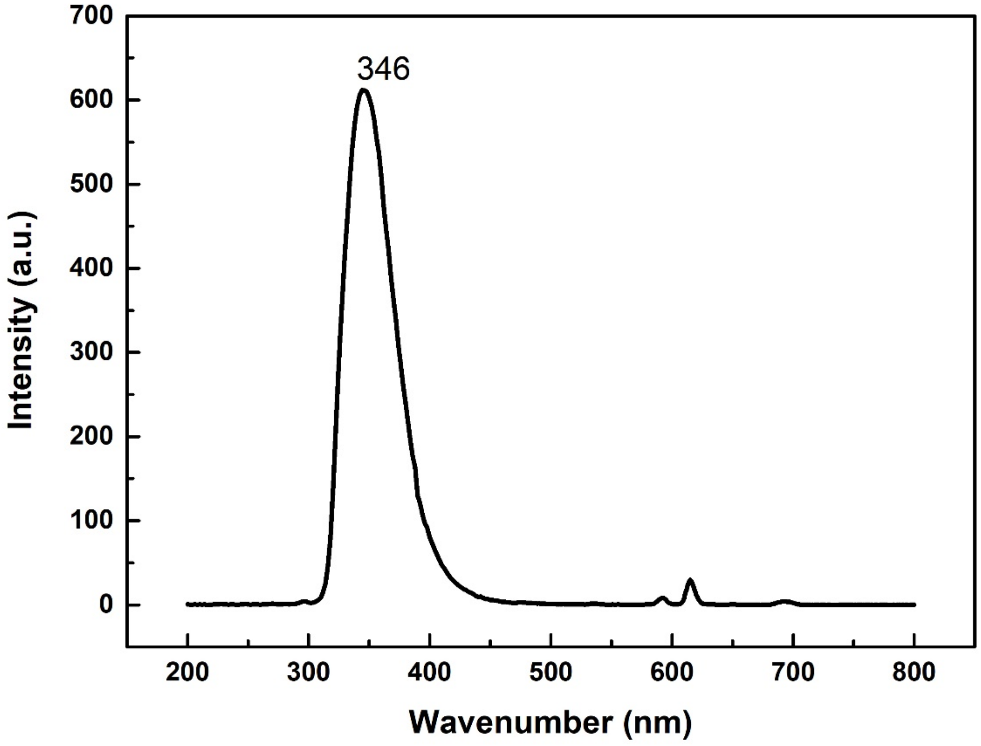

Figure S12. The luminescent emission spectra of compound **2** in DMF solution with a main emission peak at 346 nm.

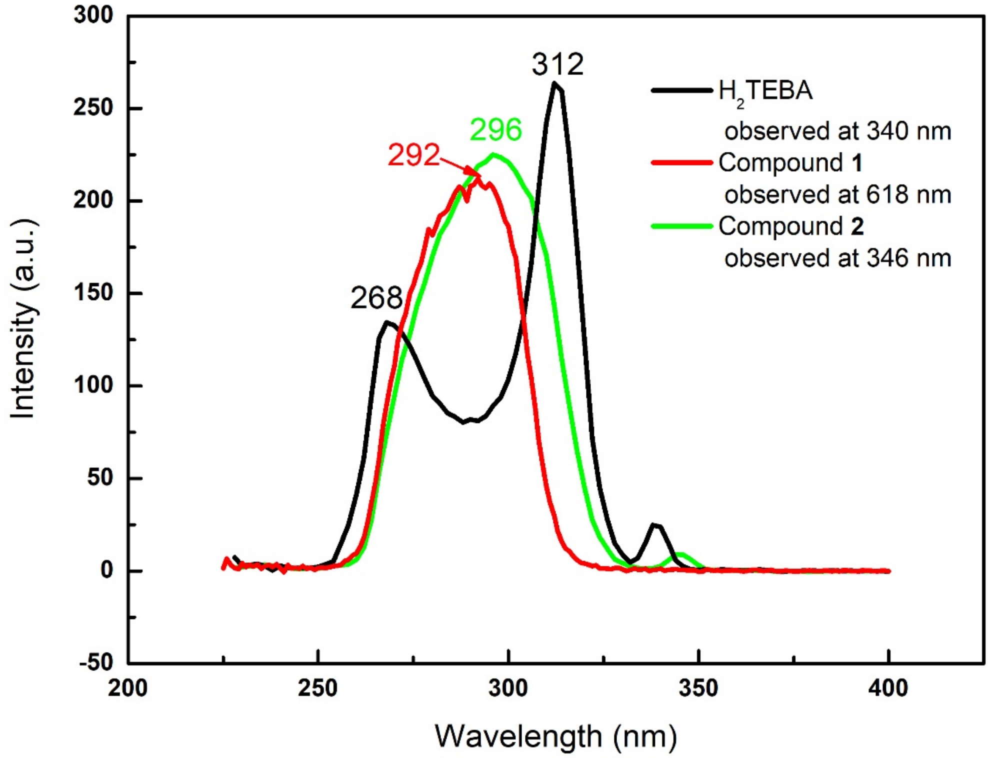

Figure S13. The excitation spectra of ligand H_2TEBA, compound **1** and compound **2** in DMF solution observed at their highest emission peak, respectively.
